# Seroprevalence of Scrub Typhus, Typhus, and Spotted Fever Among Rural and Urban Populations of Northern Vietnam

**DOI:** 10.4269/ajtmh.16-0399

**Published:** 2017-05-03

**Authors:** Nguyen vu Trung, Le Thi Hoi, Nguyen Thi Hong Thuong, Tran Khanh Toan, Tran Thi Kieu Huong, Tran Mai Hoa, Annette Fox, Nguyen van Kinh, H. Rogier van Doorn, Heiman F. L. Wertheim, Juliet E. Bryant, Behzad Nadjm

**Affiliations:** 1 National Hospital for Tropical Diseases, Hanoi, Vietnam; 2 Oxford University Clinical Research Unit and Wellcome Trust Major Overseas Programme, Hanoi, Vietnam; 3 Hanoi Medical University, Hanoi, Vietnam; 4 Department of Microbiology and Immunology, Doherty Institute for Infection and Immunity, University of Melbourne, Melbourne, Australia; 5 Center for Tropical Medicine, Nuffield Department of Clinical Medicine, University of Oxford, Oxford, United Kingdom; 6 Department of Medical Microbiology, Radboud Center for Infectious Diseases, Radboud University Medical Center, Nijmegen, The Netherlands

## Abstract

Rickettsial infections are recognized as important causes of fever throughout southeast Asia. Herein, we determined the seroprevalence to rickettsioses within rural and urban populations of northern Vietnam. Prevalence of individuals with evidence of prior rickettsial infections (IgG positive) was surprisingly low, with 9.14% (83/908) testing positive to the three major rickettsial serogroups thought to circulate in the region. Prevalence of typhus group rickettsiae (TG)–specific antibodies (6.5%, 58/908) was significantly greater than scrub typhus group orientiae (STG)– or spotted fever group rickettsiae (SFG)–specific antibodies (*P* < 0.05). The majority of TG seropositives were observed among urban rather than rural residents (*P* < 0.05). In contrast, overall antibody prevalence to STG and SFG were both very low (1.1%, 10/908 for STG; 1.7%, 15/908 for SFG), with no significant differences between rural and urban residents. These results provide data on baseline population characteristics that may help inform development of *Rickettsia* serological testing criteria in future clinical studies.

Rickettsial infections are commonly recognized as an important cause of fever and central nervous system infections throughout southeast Asia; however, there remains much debate over the relative burden of disease attributable to rickettsial agents, and many unresolved issues with diagnosis of clinical cases.^1^–^4^ Rickettsioses are zoonotic infections transmitted to humans through bites of infected fleas, mites, ticks, and lice. The epidemiology, ecology, and clinical characteristics of the rickettsioses are highly geographically variable due to species diversity of rickettsial agents as well as the diversity of arthropod vectors involved in transmission.^5^–^7^ Rickettsial agents are small Gram-negative, obligate intracellular bacteria. There are three major serogroups of rickettsial agents: scrub typhus group (STG) that includes *Orientia tsutsugamushi* and the newly described *Orientia chuto*, pathogens of scrub typhus; typhus group rickettsiae (TG) that includes *Rickettsia typhi*, the pathogen of murine typhus, and *Rickettsia prowazekii*, the agent of epidemic or louse-borne typhus which tends to be more severe than *R. typhi*; and the spotted fever group rickettsiae (SFG) that includes over 20 *Rickettsia* species worldwide. Although data from Vietnam are scant, a retrospective fever study conducted in Bach Mai Hospital, a large central hospital in Hanoi, Vietnam, found that scrub and murine typhus accounted for a surprisingly high fraction of cases of fever without focusing on other diagnoses between 2001 and 2003 (as much as 40.9% and 33% of suspect *Rickettsia* cases, respectively), when using immunofluorescence assay for confirmatory diagnosis.^8^

Diagnosis of rickettsioses is notoriously difficult for a number of reasons: 1) clinical presentations are nonspecific and highly variable (the typical eschar for scrub typhus and spotted fever is not always present and often overlooked by patients and physicians, and a rash is nonspecific and appears late in the disease); 2) the organisms are not picked up by routine culture media, and specific culture techniques mandate biosafety level 3 procedures and laboratory space; and 3) despite significant advances in diagnostic methodology in recent years, there remains a general lack of standardized and validated assays, and lack of consistent practice across different hospital clinical laboratories.^9^ An adequate understanding of local population exposure levels is important for the development of appropriate diagnostic thresholds for sample positivity, which is particularly important when only a single serum is available.

In this study, we aimed to investigate exposure levels of healthy Vietnamese people to rickettsial agents (STG, TG, and SFG), using serum collections from a previous comparative study of urban and rural residential populations in the metropolitan area of Hanoi,^10^,^11^ to support future prospective studies of rickettsial disease among suspect clinical patients. The original study that gave rise to the serum collections was implemented in 2011–2012, and entitled “Influenza and dengue transmission, and *Klebsiella pneumoniae* carriage in urban and rural sites in Ha Noi,” and took advantage of the existing framework and infrastructure of the FilaBavi demographic surveillance site.^11^ Enrollment in the influenza/dengue/*Klebsiella* study involved identifying a random selection of healthy people from two districts of Hanoi Province (urban DongDa and rural BaVi), with deliberate selection of children and young adults in view of the project emphasis on dengue. Written informed consent was obtained for sampling at two time points for nasal/throat swabs as well as serum collections, to screen for the three pathogens of interest (influenza/dengue/*Klebsiella*). The original study was approved by the Institutional Review Board of Hanoi Medical University (HMU; August 18, 2011, No. 91) and the Oxford University Tropical Research Ethics Committee (OxTREC ref. 36-11). Subsequently in 2015, to use the residual anonymized sera for additional rickettsial serologies, we obtained new approval from HMU (January 2015, ref. 165).

We used serum samples from a total of 943 individuals: 495 urban residents from Hanoi city (21.0181°N, 105.8299°E) and 448 rural residents of BaVi District, located 60 km southwest of Hanoi (between 21.0084°N and 21.3176°N and 105.2873°E and 105.4729°E), an area that includes lowland, highland, and mountainous areas, ranging in altitude from 20 to 1,297 m above sea level. Approximately 40% of participants were less than 20 years of age (median age = 32 years, range = 2–91). The age distribution was similar for both urban and rural sample sets; sampling was age stratified and designed to overrepresent children under 20 years; hence, the age distribution of samples did not reflect population structure. Unfortunately, only a single serum sample was obtained from each of the rural participants (between March and May 2012), whereas paired sera were obtained from some of the urban participants (278 pairs, collected in October 2011 and February/March 2012), for a total of 1,221 serum samples.

We conducted serological testing by enzyme-linked immunosorbent assays (ELISAs) as previously described,^12^ using antigens available from the U.S. Naval Medical Research Center, Silver Spring, MD, that have been used widely in *Rickettsia* seroprevalence studies across the globe^12^–^15^ and comprised *R. typhi* Wilmington for TG; *Rickettsia conorii* Malish 7 for spotted fever group; and *O. tsutsugamushi* Karp, Kato, and Gilliam for STG ELISA antigen preparations. Negative and positive serum controls for the assay comprised pooled samples from individuals with or without polymerase chain reaction–confirmed rickettsial infection, who had been previously diagnosed within the National Hospital of Tropical Diseases, Hanoi, and with net optical densities (ODs) of < 0.2 and > 1.0 for each antigen, respectively. For initial screening, samples were tested at 1:100 dilution and scored positive if they yielded a net OD of ≥ 0.5 for any of the three antigen preparations. All screen-positive sera were titrated at four dilutions of 1:100, 1:400, 1:1,600, and 1:6,400. Samples were considered positive when the cumulative net OD value for the four dilutions was ≥ 1,000. Titers for the positive samples were determined to be the inverse of the highest dilution that gave a net OD of > 0.2. Samples that achieved an OD > 0.5 at screening but failed to be confirmed by titration were reported as negative for all later analyses.

Of the total 943 participants, samples from 35 were excluded due to poor quality (hemolysis, lipemic, or microbial growth). Demographics and seroprevalence by antigen group of the sampled population are shown in Table 1. Overall prevalence of individuals with evidence of prior rickettsial infections (i.e., antibody positive) was low, with 9.1% (83/908) testing positive. Antibody prevalence to TG (6.5%, 58/908) was significantly greater than that to STG or SFG (*P* < 0.05), and the majority of TG seropositives were observed among urban (8.9%, 43/479) rather than rural residents (3.5%, 15/429; *P* < 0.05) (analyses by Fisher's exact test). In contrast, the overall antibody prevalence to STG and SFG were both very low (1.1%, 10/908 for STG; 1.7%, 15/908 for SFG), and there were no significant differences in exposure between rural and urban residents for either antigen, although the numbers were low. For the 15 individuals who exhibited reactivity to SFG antigen, this may constitute the first evidence of local transmission of rickettsial agents within the SFG. Of the 83 seropositive individuals, there appeared to be only low levels of multiple infections or cross-reactivity. Four people showed reactivity to more than one serogroup: one 77-year-old man from urban Hanoi had antibodies to both STG and TG, and three women from BaVi (43, 66, and 73 years) had antibodies to both STG and SFG. Among the 221 urban participants with paired sera, we detected one seroconversion (4-fold rise in titer) to TG in a 21-year-old male. Seroprevalence to each antigen group increased with age, with potential gender differences in exposure to SFG (slightly higher numbers of seropositives in males), but this did not reach statistical significance.

The findings reported here suggest that northern Vietnamese populations are not substantially exposed to rickettsial agents, and that infections largely occur in the urban environment with agents of TG, most likely *R. typhi*. We posit that murine typhus (*R. typhi*), rather than epidemic louse-borne typhus (*R. prowazekii*), is the more likely cause of the observed seropositivity, because murine typhus is a common urban disease, whereas epidemic typhus is not endemic to southeast Asia and conditions for transmission (infestations with body lice) are not highly prevalent in Vietnam. Seropositivity reported here was low compared with other studies examining population exposure levels among undifferentiated fever cases^2^,^16^–^18^ or for investigations of healthy populations in southeast Asia^13^,^15^,^19^ (Supplemental Table 1). While direct comparisons of seropositivity levels among healthy individuals versus fever patients are not valid, the overall low seropositivity detected from our cohort is surprising given the recent report by Hamaguchi and others, that indicated a large number of undiagnosed fever cases presenting to hospital in Hanoi were infected with either TG or STG. Hamaguchi and others noted that their enrollment criteria heavily selected in favor of rickettsial cases (e.g., through lack of an alternative diagnosis, presence of an eschar or nonresponse to β-lactam antibiotics) and thus may overrepresent the fraction of fever cases attributable to rickettsial agents. The low levels of seropositives detected here may indicate that the antigens used in this study were not sufficiently reactive with local circulating strains, despite our inclusion of the genotypes believed to be dominant in the region.^20^ Observed increases in age-related seropositivity suggest retention of antibodies throughout life; however, we cannot rule out the possibility that our low levels of detected IgG positivity reflect decay kinetics, such that individuals do not retain a long-term signature of prior or repeated exposures. Low levels of seropositivity may also reflect the relatively young age of the population tested. One fortunate consequence of low background antibody prevalence to rickettsial agents is that the use of unpaired ELISA serology for diagnosis of suspect cases may be less likely to yield false positives in our location. A great deal remains to be learned about the basic epidemiology, transmission, and burden of *Rickettsia* in the region; further studies to investigate seroprevalence in both clinical populations as well as healthy individuals are needed to assess potential risks to human populations, and to forecast how changing patterns of land use and urbanization may impact spread of disease.

## Supplementary Material

Supplemental Table.

## Figures and Tables

**Table 1:** Descriptive data on the serum sample set and seropositivity against the three test rickettsial antigen preparations

Age group	Total no. sera	No. pair sera	Median age (years)	Female gender (%)	No. Dong Da (Urban)	No. BaVi (Rural)	Number seropositives (%)		
							STG	SFG	TG
< 5	41	6	4	53.7	21	20	0	0	0
5–12	208	46	8	49	87	121	0	0	0
13–26	183	52	16	50.3	112	71	2 (1.9%)	0	10 (5.5%)
> 26	476	137	51	59	259	217	8 (1.7%)	15 (3.2%)	48 (10.1%)
Overall	908	241	29	54.7	479	429	10 (1.1%)	15 (1.7%)	58 (6.5%)

SFG = spotted fever group rickettsiae; STG = scrub typhus group orientiae; TG = typhus group rickettsiae.

## References

[1] Acestor N, Cooksey R, Newton P, Menard D, Guerin PJ, Nakagawa J, Christophel E, Gonzalez IJ, Bell D (2012). Mapping the aetiology of non-malarial febrile illness in southeast Asia through a systematic review: terra incognita impairing treatment policies. PLoS One.

[2] Aung AK, Spelman DW, Murray RJ, Graves S (2014). Rickettsial infections in southeast Asia: implications for local populace and febrile returned travelers. Am J Trop Med Hyg.

[3] Richards AL (2012). Worldwide detection and identification of new and old rickettsiae and rickettsial diseases. FEMS Immunol Med Microbiol.

[4] Dittrich S, Rattanavong S, Lee SJ, Panyanivong P, Craig SB, Tulsiani SM, Blacksell SD, Dance DAB, Dubot-pérès A, Sengduangphachanh A, Phoumin P, Paris DH, Newton PN (2015). *Orientia*, *Rickettsia*, and *Leptospira* pathogens as causes of CNS infections in Laos: a prospective study. Lancet Glob Health.

[5] Dumler JS, Barbet AF, Bekker CP, Dasch GA, Palmer GH, Ray SC, Rikihisa Y, Rurangirwa FR (2001). Reorganization of genera in the families Rickettsiaceae and Anaplasmataceae in the order Rickettsiales: unification of some species of *Ehrlichia* with *Anaplasma*, *Cowdria* with *Ehrlichia* and *Ehrlichia* with *Neorickettsia*, descriptions of six new species combinations. Int J Syst Evol Microbiol.

[6] Elisberg B, Campbell J, Bozeman F (1968). Antigenic diversity of *Rickettsia tsutsugamushi*: epidemiologic and ecologic significance. J Hyg Epidemiol Microbiol Immunol.

[7] Azad AF (1990). Epidemiology of murine typhus. Annu Rev Entomol.

[8] Hamaguchi S, Cuong NC, Tra DT, Doan YH, Shimizu K, Tuan NQ, Yoshida L-M, Mai LQ, Duc-Anh D, Ando S, Arikawa J, Parry CM, Ariyoshi K, Thuy PT (2001). Clinical and epidemiological characteristics of scrub typhus and murine typhus among hospitalized patients with acute undifferentiated fever in northern Vietnam. Am J Trop Med Hyg.

[9] Lim C, Paris DH, Blacksell SD, Laongnualpanich A, Kantipong P, Chierakul W, Wuthiekanun V, Day NPJ, Cooper BS, Limmathurotsakul D (2015). How to determine the accuracy of an alternative diagnostic test when it is actually better than the reference tests: a re-evaluation of diagnostic tests for scrub typhus using Bayesian LCMs. PLoS One.

[10] Dao TT, Liebenthal D, Tran TK, Ngoc Thi Vu B, Ngoc Thi Nguyen D, Thi Tran HK, Thi Nguyen CK, Thi Vu HL, Fox A, Horby P, Van Nguyen K, Wertheim HFL (2014). *Klebsiella pneumoniae* oropharyngeal carriage in rural and urban Vietnam and the effect of alcohol consumption. PLoS One.

[11] Chuc N, Diwan V (2003). FilaBavi, a demographic surveillance site, an epidemiological field laboratory in Vietnam. Scand J Public Health Suppl.

[12] Graf PCF, Chretien J-P, Ung L, Gaydos JC, Richards AL (2008). Prevalence of seropositivity to spotted fever group rickettsiae and *Anaplasma phagocytophilum* in a large, demographically diverse US sample. Clin Infect Dis.

[13] Richards AL, Soeatmadji D, Widodo M, Sardjono T, Yanuwiadi B, Hernowati TE, Baskoro AD (1997). Seroepidemiologic evidence for murine and scrub typhus in Malang, Indonesia. Am J Trop Med Hyg.

[14] Thiga JW, Mutai BK, Richards AL, Waitumbi JN (2015). High seroprevalence of antibodies against in patients with febrile illness, Kenya. Emerg Infect Dis.

[15] Vallée J, Thaojaikong T, Moore CE, Phetsouvanh R, Richards AL, Souris M, Fournet F, Salem G, Gonzalez JPJ, Newton PN (2010). Contrasting spatial distribution and risk factors for past infection with scrub typhus and murine typhus in Vientiane city, Lao PDR. PLoS Negl Trop Dis.

[16] Blacksell SD, Kantipong P, Watthanaworawit W, Turner C, Tanganuchitcharnchai A, Jintawon S, Laongnuanutit A, Nosten FH, Day NPJ, Paris DH, Richards AL (2015). Underrecognized arthropod-borne and zoonotic pathogens in northern and northwestern Thailand: serological evidence and opportunities for awareness. Vector Borne Zoonotic Dis.

[17] Maude RR, Maude RJ, Ghose A, Amin MR, Islam MB, Ali M, Bari MS, Majumder MI, Tanganuchitcharnchai A, Dondorp AM, Paris DH, Bailey RL, Faiz MA, Blacksell SD, Day NP (2014). Serosurveillance of *Orientia tsutsugamushi* and *Rickettsia typhi* in Bangladesh. Am J Trop Med Hyg.

[18] Tay S, Kamalanathan M, Rohani M (2003). Antibody prevalence of *Orientia tsutsugamushi*, *Rickettsia typhi* and TT118 spotted fever group rickettsiae among Malaysian blood donors and febrile patients in the urban areas. Southeast Asian J Trop Med Public Health.

[19] Strickman D, Tanskul P, Eamsila C, Kelly D (1994). Prevalence of antibodies to rickettsiae in the human population of suburban Bangkok. Am J Trop Med Hyg.

[20] Duong V, Mai TTX, Blasdell K, Lo LV, Morvan C, Lay S, Anukool W, Wongprompitak P, Suputtamongkol Y, Laurent D, Richner B, Ra C, Chien BT, Frutos R, Buchy P (2013). Molecular epidemiology of *Orientia tsutsugamushi* in Cambodia and central Vietnam reveals a broad region-wide genetic diversity. Infect Genet Evol.

