# Supplementary material for: Seroprevalence of Scrub Typhus, Typhus, and Spotted Fever Among Rural and Urban Populations of Northern Vietnam
Source: Am J Trop Med Hyg. 2017 May 3;96(5):1084–7. doi: 10.4269/ajtmh.16-0399 (PMC5417199; doi:10.4269/ajtmh.16-0399)
Supplement: Supplementary file 1 [file SD2.pdf]

Supplemental Table 1

Seroprevalence data reported from various studies that used ELISAs or indirect IPP assays similar to the current study

Country	% STG positive	% TG positive	% SFG positive	Cutoff	Notes	Reference
Bangladesh	66 (IgM)	23 (IgM)	ND	Single dilution, 1:100; net OD ≥ 1.0	Residual sera collections from hospitals; all ages; no exclusions	Maude and others, 2014
Kenya	5	< 1	10	Screening at 1:100 followed by titrations to confirm; cumulative OD ≥ 1.0	*N* = 2,225; febrile patients, all ages, mean 5 years	Thiga and others, 2014
Lao People's Democratic Republic	20.3	20.6	ND	For STG, used a commercial PanBio kit; for TG, used similar antigens as Thiga	*N* = 2,002; healthy adults ≥ 35 years	Vallee and others, 2010
Indonesia	1.3	34.7	ND	Titration from 1:100 to 1:6,400; highest titer with net OD ≥ 0.2	*N* = 464 healthy individuals, all ages; mean 34 years	Richards and others, 1997
Thailand*	21 (IgM)	8 (IgM)	4 (IgM)	Screening at 1:50, followed by endpoint titrations	*N* = 215 healthy individuals, all ages	Strickman and others, 1994
Malaysia*	8.8	1.7	3.7	Titrations 1× to 1:1,600; reactivity at 1:100 reported here	*N* = 295 febrile inpatients, all ages	Tay and others, 2003
Thailand	ND	ND	30	Single dilution, 1:100; "index calibrator" ≥ 1.2	*N* = 375 febrile patients; ≥ 15 years age	Blacksell and others, 2015
United States			6	Same as Thiga (except no screening, all samples were titrated)	*N* = 10,000; military personnel, all deployments; mean age 28 years	Graf and others, 2014
Vietnam	1.1	6.5	1.7	Same as Thiga		Our study

ELISA = enzyme-linked immunosorbent assay; IPP = immunoperoxidase; OD = optical density; ND = not done; SFG = spotted fever group rickettsiae; STG = scrub typhus group orientiae; TGR = typhus group rickettsiae. Unless otherwise indicated, the reported serologies refer to IgG analyses.

*Testing performed by indirect IPP assay.
